# Screen Time and Bone Status in Children and Adolescents: A Systematic Review

**DOI:** 10.3389/fped.2021.675214

**Published:** 2021-12-01

**Authors:** Carmela de Lamas, Paula Sánchez-Pintos, María José de Castro, Miguel Sáenz de Pipaon, María Luz Couce

**Affiliations:** ^1^Santiago de Compostela University, Santiago de Compostela, Spain; ^2^Metabolic Unit, Neonatology Department, University Clinical Hospital of Santiago de Compostela, Santiago de Compostela, Spain; ^3^IDIS-Health Research Institute of Santiago de Compostela, Santiago de Compostela, Spain; ^4^European Reference Network for Rare Hereditary Metabolic Disorders (MetabERN), Madrid, Spain; ^5^Rare Diseases Networking Biomedical Research Centre (CIBERER), Madrid, Spain; ^6^Department of Pediatrics-Neonatology, Autonomous University of Madrid, La Paz University Hospital, Madrid, Spain

**Keywords:** bone mineral density, computer, mobile phone, screen, tablets, bone turnover

## Abstract

**Introduction:** Technological advances over the last 2 decades have led to an increase in the time spent by children and youth engaged in screen-based activities, and growing recognition of deleterious effects on health. In this systematic review of cohort and cross-sectional studies, we assess current data on the relationship between screen time and bone status in children and teenagers.

**Methods:** We searched PUBMED and SCOPUS databases for studies of children and adolescents that assessed screen time and bone status, determined by measuring bone mineral content or density, bone stiffness index, bone speed of sound, bone broadband ultrasound attenuation, or frame index. Searches were limited to studies published between 1900 and 2020, and performed in accordance with Preferred Reporting Items for Systematic Review and Meta-Analyses (PRISMA) guidelines. The studies included were evaluated using the Newcastle-Ottawa quality assessment scale.

**Results:** Ten cohort and cross-sectional studies including pediatric population were selected. The combined study population was 20,420 children/adolescents, of whom 18,444 participated in cross-sectional studies. Four studies assessed the effects of total screen time, seven the consequences of TV viewing time, and six the effects of recreational computer use on bone health. Our findings indicate an inverse association between total and weekly screen time and bone health in children and adolescents. In 57% of the studies included also a negative correlation between television viewing time and bone status was observed, while recreational computer time did not have a significant impact on bone health. According to the only four studies that included dietetic factors, no relevant differences were found between calcium intake and screen time or bone broadband ultrasound attenuation and bone speed of sound.

**Conclusions:** Review of the literature of the past three decades provides strong support for comprehensive education of screen time on bone status. The findings of this systematic review support a negative association between screen time and bone status in children and adolescents, with a different impact when considering the different technological devices. As peak bone mass in adolescents is the strongest predictor of osteoporosis risk, strategies aimed at improving bone health should incorporate conscious use of digital technology.

## Introduction

In recent years screen use has expanded to include a wide variety of electronic media devices available throughout the world. Although television (TV) remains the predominant screen-based activity among children (1), use of computers, video games, tablets, and smart phones begins at increasingly younger ages (2–4). The popularity and widespread use of screen-based activities among children and young people and the accompanying rapid change in technology and patterns of use, has turned the detrimental effects of excessive screen time and its prevalence into a global health problem. A recent study that compared screen time exposure in young children before and after mobile devices became widely available found that between 1997 and 2014 total screen time in children aged 0–2 years increased from 1.32 h to 3.05 h per day, and that most of this time was spent watching TV (5). Since the invention of television, parents, educators, and health care providers have raised concerns about the immediate and long-term deleterious effects of excessive screen-based activity, especially TV viewing (6–9). Several studies have reported negative associations between screen time and physical and cognitive abilities (10), and positive associations with obesity (11), sleep problems, attention disorders, depression, and anxiety (12–14). Excessive screen exposure can also cause visual discomfort (15, 16), myopia, or squinting due to a lack of outdoor activities (17), and video games and TV viewing in particular are associated with unhealthy diets (18). Other concerns relate to the exposure of children to potentially deleterious content, including violence, sex, and fast food advertising (19).

The American Academy of Pediatrics (AAP) has recommended limiting children's total media time (with entertainment media) to no more than 1–2 h of quality programming per day (20), no screen time for children under 2 years of age, and removal of TV sets from children's bedrooms. Several studies have investigated the individual, familial, and sociocultural forces that shape children's screen habits to identify simple and incremental approaches that may help reduce TV viewing time (21–23). Although most parents report that they adhere to TV viewing guidelines, few establish rules that limit the time their children spend watching TV (24, 25). Moreover, while parents tend to agree with a 2-h limit in principle, many feel that it does not apply to their child in the absence of academic difficulties or behavioral problems, and perceive numerous barriers to implementing the recommendations (26).

It is increasingly acknowledged that screen-based activity may also negatively affect bone status, resulting in low bone mineral content (BMC), low bone mineral density (BMD), and osteoporosis (27), since nutrition (including adequate intake of protein, Calcium (Ca), Phosphorus (P), and vitamin D) (28, 29) and physical activity are major factors implicated in bone growth and health. In general, dual energy X-ray absorptiometry (DEXA) of the lumbar spine and hip is the preferred method of measuring BMD. The International Society for Clinical Densitometry (ISCD) recommends using DEXA BMD Z-scores rather than T-scores in children, since diagnosis of osteoporosis in these groups should not be based on densitometry criteria alone and should include the presence of a clinically significant fracture history (30). While the current gold standard for measuring BMD is DEXA, this method is costly, involves ionizing radiation, and requires a highly trained operator (31). Another method developed to assess osteoporosis risk is quantitative ultrasound (QUS) (17). QUS assesses bone quality by measuring the attenuation and velocity of ultrasound waves passing through the bone, and has become a popular low-cost, readily accessible, and radiation-free alternative to DEXA for osteoporosis screening (32, 33).

The incidence, severity, underlying mechanisms, and clinical implications of bone disease associated with screen use in children remain a matter of discussion. In this systematic review we present a comprehensive overview of evidence from cohort and cross-sectional studies assessing the association between screen-based activity and bone status in children and adolescents, including BMC, BMD, bone stiffness index (BSI), bone speed of sound (SOS), bone broadband ultrasound attenuation (BUA), and frame index (FI).

## Methods

The review question on which this work was based was as follows: “Does screen time in children and adolescents correlate with bone status?”. This systematic review was carried out following PRISMA (Preferred Reporting Items for Systematic reviews and Meta-analyses) guidelines (34, 35) and was registered in the International Prospective Register of Systematic Reviews (PROSPERO Code: CRD42020217924. Data registry: Nov 28, 2020).

Once the review question was formulated, we performed searches of PUBMED and SCOPUS databases in December 2020. The PUBMED search was carried out using the following search terms: (“Bone and Bones”[Mesh] OR “Calcification, Physiologic”[Mesh]) AND (“Life Style”[Mesh] OR “Sedentary Behavior”[Mesh] OR “television”[Mesh] OR “screen time”[Mesh]). The search was limited to the pediatric population through the filter: Child: birth-18 years. The SCOPUS search was conducted using the search terms “(Television OR screen time) AND Bone”. In addition, the bibliographies of the articles returned by searches and other previously published reviews on the topic were manually reviewed.

Based on the PICOS criteria (Population, Intervention, Comparison, Outcome and, Settings) (34) the inclusion criteria were: children and adolescents, assessing screen time (TV, computer, mobile devices) and bone status observational studies published between January 1, 1900 and December 31, 2020. Studies that did not include screen time or bone status data or were carried out in patients with chronic pathologies including obesity, single case review studies or studies written in languages other than Spanish and Englis were excluded from our review.

The time dedicated to screen-based activities was the exposure studied. The purpose of the study was to assess how this variable influences bone health in children and adolescents. Studies that fulfilled the inclusion criteria, regardless of the number (although always >1) and ethnicity of participants and the duration of exposure, were eligible for inclusion in the review process. Potential confounding factors related to diet such as calcium and protein intake, soft drinks and dairy consumption were also included when data were available.

Two types of outcome measures were considered useful for evaluating bone status: BMC and BMD.

Results considered valid were those that included BMC or BMD measurements taken using DEXA and reported as absolute values or z-scores for the whole body, lumbar spine, femoral neck, or extremities; skeletal robustness (BSI measured in the calcaneus bone); SOS measured in the radius, tibia, or calcaneus; BUA measured in the calcaneus by quantitative ultrasound; and the anthropometric index FI [(elbow breadth in mm/ (height in cm) × 100]. Non measurable-data, such as evaluation of deformities on radiographs, were excluded.

The 10 studies finally included were selected independently by two authors from the 414 articles identified during the bibliographic search. In cases in which there was a lack of consensus the remaining authors acted as arbitrators.

Two authors independently collected data from the articles considered for review. The following data were extracted from each study: number, age and sex of participants, type of study, outcome measures, results, and conclusions. The remaining authors arbitrated in cases in which any discrepancies arose.

The risk of bias assessment was performed using the Newcastle-Ottawa quality assessment scale (36). This scale studies the risk of bias during participant selection, comparison between individuals, and exposure assessment. Based on the analysis of each of these risks each article is awarded a maximum of 9 stars, corresponding to the selection process (maximum, 4 stars), comparability between groups (maximum, 2 stars); and exposure assessment (maximum, 3 stars). Seven or more stars are considered indicative of a good quality study.

## Results

**Figure 1:**
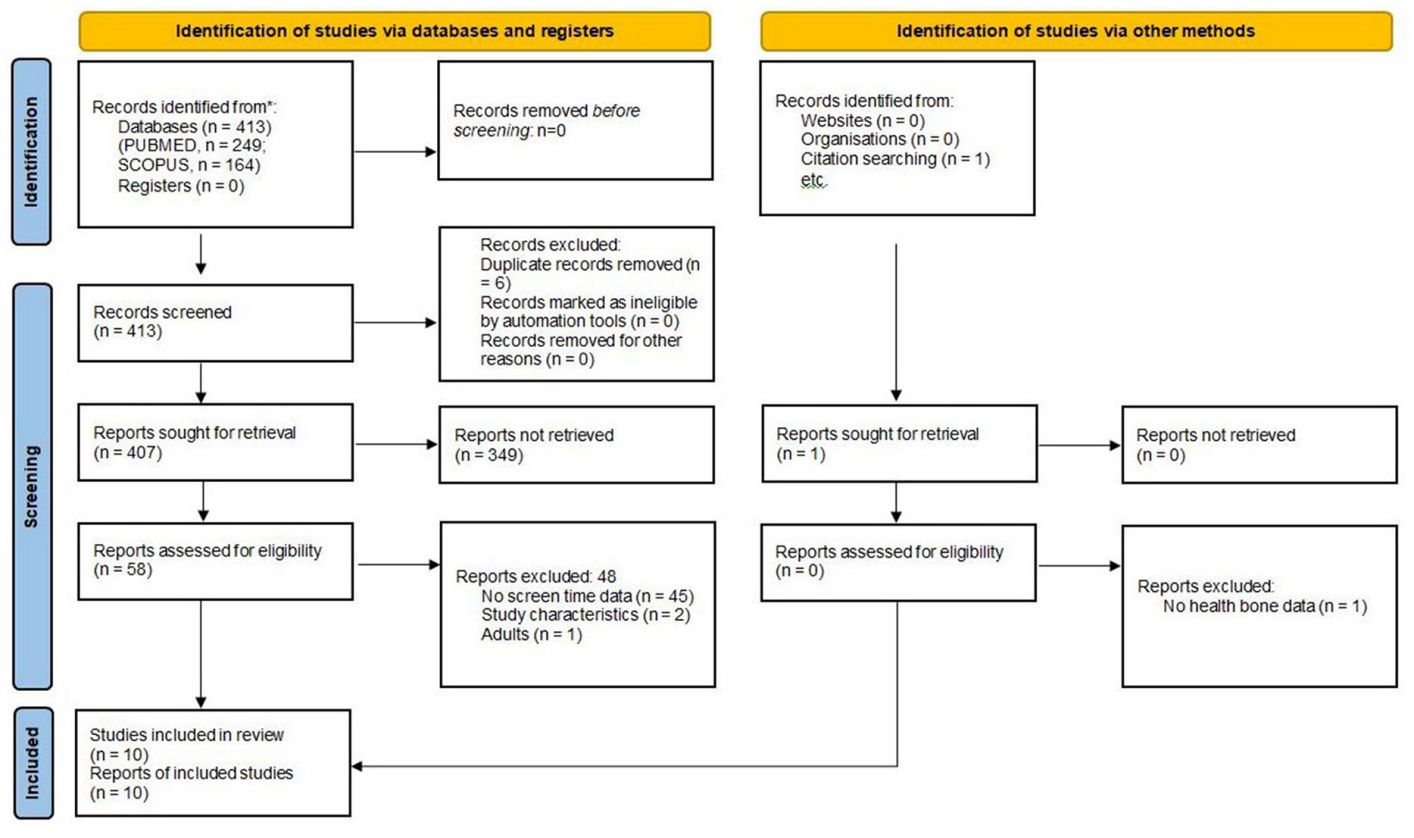
PRISMA 2020 flow diagram for systematic reviews which included searches of databases, registers, and other sources.

The process by which articles were selected for this systematic review is summarized in Figure 1. The SCOPUS search returned 164 articles, while the PUBMED search identified a further 249 studies. One other article, identified in the manual review of the bibliography of the aforementioned articles, was also included. Of the 414 articles found in database searches, 6 duplicate articles were excluded, and 349 were excluded due to a lack of relevance of the abstract (129 lacked screen time data, 106 lacked bone health data, 65 were studies of adult populations, 27 recruited unhealthy individuals, 21 were narrative reviews, and 1 was a preclinical study). Of the 59 full-text articles reviewed, 45 were excluded due to a lack of screen time data; 2 due to unsuitable study characteristics; 1 due to the absence of bone health data; and 1 because the study population was exclusively adult (Supplementary Table 1). Ultimately, 10 articles (37–46) were selected for inclusion in this systematic review.

### Study Characteristics

Tables 1–3 summarize the main characteristics of the 10 selected cohort and cross-sectional studies, which are ordered according to the age of the study population. Three cohort studies were included (40, 45, 46). All studies were published after 2004, and 4 in the last 5 years (37, 38, 41, 42). The combined study population of the 10 studies was 20,420 children and adolescents, of whom 18,444 participated in cross-sectional studies. The age of the study populations ranged from 2–22 years. All studies involved a pediatric population (range, 2–18 years) and 1 also included young adults up to 22 years of age (37). Four studies assessed the effects of total screen time on bone health (37–40), 7 the effects of TV viewing time on bone health (37, 39–44), and 6 the effects of recreational computer use on bone health (39, 41, 42, 44–46). Four studies used DEXA to evaluate bone health (38, 39, 42, 44), of which 2 measured BMC (39, 44) and the other 2 measured BMD (38, 42). Six studies used QUS (37, 40, 41, 43, 45, 46) to measure BSI (2 studies) (37, 41), SOS (3 studies) (40, 45, 46), FI (1 study) (43), or BUA (2 studies) (45, 46). In the selected studies screen time was measured as *TV and/or computer time* in hours/week in 4 studies (37, 41, 45, 46) and in minutes or hours/day in 6 studies (38–40, 42–44). One study (42) also analyzed *videogame time* in minutes/day and internet use for non-school purposes in minutes/day.

**Table 1:** Effects of total screen time on bone health.

**Reference**	**Study design**	***n***	**Age, y^**1**^**	**Dietetic factors**	**Outcome measure**	**Results^**2**^**	**Conclusions**
Herrmann et al. (37)	Cross sectional	PSC, 1512 SC, 2953	PSC, 2–5 SC, 6–10	Dairy products consumption (frequency/week): PSC, 21+-12 SC, 20+-12	Association between self-reported screen time (hours/week) and BSI in calcaneus (QUS, multivariate linear regression)	PSC,−0.07; SC, 0.002	No significant association
Winther et al. (38)	Cross sectional	9551 (469 F)	16.6 ± 0.41	Sufficient calcium intake (%): 0–2 h/d: B, 91.8; G, 89 2–4 h/d: B, 92.9; G, 89.6 4–6 h/d: B, 90.8; G, 87.9 ≥6 h/d: B, 87.1; G, 77.8 Soft drinks consumption (%): 0-2 h/d: Rarely B, 14.3; G, 52.1; 1 glass a day B, 67.3, G, 46.6; ≥ 2 glasses a day B 16.3; G, 1.4. 2-4 h/d: Rarely B, 17; G, 37; 1 glass a day B, 67; G, 37. ≥ 2 glasses a day B 16.1; G, 1.9. 4-6 h/d: Rarely B, 5.4; G, 28.3; 1 glass a day B,79.3; G, 65.3; ≥ 2 glasses a day B 15.2; G, 5.8. ≥6 h/d: Rarely B, 7.2; G, 22.2; 1 glass a day B, 66.9; G, 57.1; ≥2 glasses a day B, 25.2; G, 17.5.	Association between self-reported screen-based sedentary activity time on weekends and BMD in g/cm^2^ in hip, femoral neck, and total body (DEXA)	Hip BMD 2–4 h/d: B, −0.061 [−0.111; 0.011]; G, 0.025 [−0.008; 0.059]; 4–6 h/d: B, −0.038 [−0.087; 0.011]; G, 0.054 [0.017; 0.09]; ≥6 h/d: B, −0.062 [−0.120; 0.004]; G, 0.042 [−0.006; 0.09] Femoral Neck BMD 2–4 h/d: B, −0.063 [−0.113; 0.014]; G, 0.046 [0.012; 0.079]; 4–6 h/d: B, −0.034 [−0.083; 0.014]; G, 0.07 [0.034; 0.106];≥6 h/d: B, −0.064 [−0.121; −0.007]; G, 0.058 [0.01; 0.105] Total body BMD 2–4 h/d: B, −0.039 [−0.068; −0.01]; G, 0.015 [−0.003; 0.033]; 4–6 h/d: B, −0.028 [−0.056; 0.001]; G, 0.023 [0.003; 0.042]; ≥6 h/d: B, −0.03 [−0.064; 0.004]; G, 0.017 [−0.009; 0.043]	Weekend screen time is inversely associated with BMD levels in boys
Chastin et al. (39)	Cross sectional	1348 (677 F)	8–22	No data	Association between self-reported screen time and BMC in g in proximal femur and lumbar L1-L4 spine (DEXA)	Femoral BMC: B, −0.21 [−0.41; 0.00]; G, −0.8 [−1.35; −0.25] Spinal BMC: B, −1.17 [−2.6; 0.25]; G, −0.4 [−0.66; −0.18]	Total screen time is negatively associated with femoral BMC in boys and girls and with spinal BMC in girls only
Christoforidis et al. (40)	Cohort study	1549 (814 F)	11.41 ± 3.52 (3.78–18.33)	No data	Association between self- reported total daily screen time and SOS in m/s at radius and tibia (QUS)	SOS at radius: ≤ 1 h/d, 0.02 ± 1.04; 1–3 h/d, 0.05 ± 0.94;3–5 h/d −0.07 ± 1.07; >5 h/d, −0.19 ± 0.94 SOS at tibia: ≤ 1 h/d, 0.01 ± 0.97; 1–3 h/d, 0.05 ± 1.38;3–5 h/d, −0.06 ± 0.99; >5 h/d, −0.126 ± 1.05	Total daily screen time is associated with a significant decrease in SOS values in radius and tibia

*B, boys; BMC, bone mineral content; BMD, bone mineral density; BSI, bone stiffness index; d, day; DEXA, dual energy x-ray absorptiometry; F, female; G, girls; h, hours; PSC, preschool children group; QUS, quantitative ultrasound; SC, school children group; SOS, speed of sound; y, years*.

1 * Values represent the range or the mean ± SD in years, as reported in the corresponding article*.

2 * Values represent mean ± SD; β (95%CI) or β as reported in the corresponding article*.

**Table 2:** Effects of television viewing time on bone health.

**Reference**	**Study design**	***n***	**Age, y^**1**^**	**Dietetic factors**	**Outcome measure**	**Results^**2**^**	**Conclusions**
Cheng et al. (41)	Cross sectional	2008(922 F)	6.14 ± 1.8	No data	Association between self-reported TV hours/ week and BSI in calcaneus (QUS)	Calcaneus BSI: NWG, −0.35 [−0.69; 0.01]. OBG, 0.03 [−0.6; 0.66]	Inverse association between weekly TV viewing time and BSI percentiles in NWG
Pelegrini et al. (42)	Cross sectional	104	10.0–14.9	No data	Association between self-reported weekly TV viewing time and total and lumbar BMD and BMC in g (DEXA)	Total body BMD: NWG, 0.031; OBG, −0.049 Lumbar BMD: NWG, 0.039; OBG, 0.041 Total body BMC: NWG, −0.012; OBG, −0.042 Lumbar BMC: NWG, 0.027; OBG, 0.062	No significant association
Chastin et al. (39)	Cross sectional	1348 (677 F)	8–22	No data	Association between self-reported TV viewing time and BMC in g in proximal femur and lumbar L1-L4 spine (DEXA)	Femoral BMC: B, −0.44 [−0.84; −0.05]. G, −0.28 [−0.5; −0.06] Spinal BMC: B, −0.47 [−1.06; 0.12].G, −0.49 [−0.95; −0.02]	Negative association between TV viewing time and femoral BMC in boys and girls and with spinal BMC in girls only
Rietsch et al. (43)	Cross sectional	691	6–10	No data	Correlation between daily self-reported TV viewing time and Frame Index	Frame index correlation: 0.063 (p=0.118)	No significant association
Vicente-Rodríguez et al. (44)	Cross sectional	277 (168 F)	13.0–18.5	No data	Risk of low BMC (DEXA) related to self-reported TV viewing time	OR low BMC: B, 7.01 [1.73; 28.4]. G, 1.26 [0.33; 4.77]	TV viewing time ≥3 h/day associated with an increased risk of low BMC in males
Babaroutsi et al. (45)	Cohort study	192(0 F)	11.9 ± 1.81	Carbohydrate (g/day and g/1000Kcal per day): 236.7 ± 98.6; 109.1 ± 23.3 Protein intake (g/day and g/1000Kcal per day):79.2 ± 31.1; 37.3 ± 10.3. Ca intake (mg/day and mg/1000Kcal per day):1039 ± 523; 487 ± 188	Association between self-reported TV viewing time and SOS and BUA in mid calcaneus	Data not shown	No significant association
Babaroutsi et al. (46)	Cohort study	217 (217 F)	12.0 ± 1.2	Carbohydrate intake (%):43.9 ± 8.7 Protein intake (%): 14.9 ± 3.9 Ca intake (mg/day and mg/1000Kcal per day): 924± 459; 523 ± 210	Association between self-reported TV viewing time and SOS and BUA in mid calcaneus	Data not shown	No significant association

*B, boys; BMC, bone mineral content; BSI, bone stiffness index; BUA, broadband ultrasound attenuation; Ca, Calcium; CG, control group; DEXA, dual energy x-ray absorptiometry; F, female; G, girls; NWG, normal weight group; OBG, obese group; QUS, quantitative ultrasound; SOS, speed of sound in m/s; TV, television; y, years*.

1 *Values represent the range, the mean (range) or the mean ± SD in years, as reported in the corresponding article*.

2 *Values represent the mean ± SD, Rho (Spearman correlation), OR (95%CI), β (95% CI) or β [99%CI] as reported in the corresponding article*.

**Table 3:** Effects of recreational computer usage time on bone health.

**Reference**	**Study design**	***n***	**Age, y^**1**^**	**Dietetic factors**	**Outcome measure**	**Results^**2**^**	**Conclusions**
Cheng et al. (39)	Cross sectional	2008 (922 F)	6.14 ± 1.8	No data	Association between self-reported hours of computer/videogames per week and BSI in calcaneus (QUS)	Calcaneus BSI: NWG, 0.03 [−0.52; 0.58] OBG, 0.03 [−0.96; 1.01]	No significant association
Pelegrini et al. (42)	Cross sectional	104 52 NWG 52 OBG	10.0–14.9	No data	Association between self- reported weekly time spent on videogames, computer games, and internet for non-school purposes and total and lumbar BMD and BMC in g (DEXA)	VG Total body BMD: NWG, −0.018; OBG, −0.135 Lumbar BMD: NWG, −0.074; OBG, −0.170 Total body BMC: NWG, 0.022; OBG, −0.154Lumbar BMC: NWG, 0.02; OBG, −0.079 CG Total body BMD: NWG, −0.085; OBG, −0.130 Lumbar BMD: NWG, −0.305; OBG, −0.097 Total body BMC: NWG, −0.097; OBG, −0.163 Lumbar BMC: NWG, −0.162; OBG, −0.138 IU Total body BMD: NWG, 0.275; OBG, 0.319 Lumbar BMD: NWG, 0.373; OBG, 0.313 Total body BMC: NWG, 0.272; OBG, 0.345 Lumbar BMC: NWG, 0.366; OBG, 0.294	Positive relationship between use of the internet for non–school purposes and total and lumbar BMD, and with lumbar BMC (and total BMC in overweight group only). Negative correlation between computer use and lumbar BMD in normal weight group.
Chastinet al. (39)	Cross sectional	1348 (677 F)	8–22	No data	Association between self-reported time spent on computer and BMC in g (DEXA) in proximal femur and lumbar L1-L4 spine	Femoral BMC: B, −0.41 [−0.90; 0.14]; G, −0.18 [−0.54; 0.19] Spinal BMC: B, −0.51 [−1.32; 0.31]; G, −0.53 [−1.29; 0.29]	No significant association
Vicente-Rodríguez et al. (44)	Cross sectional	277 (168 F)	13.0–18.5	No data	Risk of low BMC (DEXA) related to time spent on video games	OR of low BMC Video game school day: 1.66 [0.32; 8.62] Video game weekend day: 2.44 [0.75; 7.92] Video game whole week: 1.43 [0.87; 2.34]	No significant association
Babaroutsi et al. (45)	Cohort study	192 (0 F)	11.9 ± 1.81	Carbohydrate (g/day and g/1000Kcal per day): 236.7 ± 98.6; 109.1 ± 23.3 Protein intake (g/day and g/1000Kcal per day): 79.2 ± 31.1; 37.3 ± 10.3Ca intake (mg/day and mg/1000Kcal per day): 1039± 523; 487 ± 188	Association between self-reported recreational computer use and SOS and BUA in mid calcaneus	Data not shown	No significant association
Babaroutsi et al. (46)	Trial Type	217 (217 F)	12.0 ± 1.2	Carbohydrate intake (%):43.9 ± 8.7 Protein intake (%):14.9 ± 3.9 Ca intake (mg/day and mg/1000Kcal per day): 924± 459; 523 ± 210	Association between self-reported recreational computer use and SOS and BUA in mid calcaneus	Data not shown	No significant association

*B, boys; BMD, bone mineral density; BSI, bone stiffness index; BUA, broadband ultrasound attenuation; Ca, Calcium; CG, computer games; DEXA, dual energy x-ray absorptiometry; F, female; G, girls; IU, internet use; NWG, normal weight group; OBG, obese group; QUS, quantitative ultrasound; SOS, speed of sound; VG, videogames; y, years*.

1 *Values represent the range, the mean [range] or the mean ± SD in years, as reported in the corresponding article*.

2 *Values represent the mean ± SD or β (99%CI) as reported in the corresponding article*.

### Dietetic Factors

Only four studies (40%) (37, 38, 45, 46) included dietetic factors. Three of them assessed calcium intake (38, 45, 46) as sufficient calcium intake (%) (38) or absolute calcium intake (mg/day and mg/1000Kcal/day) (45, 46). No significant difference between calcium intake and screen time (38) or BUA and SOS (45, 46) was found in any of them. One included dairy products consumption (frequency/week) (37) and did not find any significant difference between preschool and school children and adolescent group but they did not assess its relation with bone status. One assessed soft drinks consumption (38) in glasses/day according to screen time during weekends, founding a significant positive relation in both sexes. Protein and carbohydrate intake (% and g/day) was assessed in males and females by Babaroutsi et al. (45, 46), and they did not found a significant difference when correlated with BUA and SOS.

### Total Screen Time and Bone Health

Four articles included in this systematic review assessed the effects of total screen time on bone health (37–40). Three of the studies were cross-sectional (37–39) and 1 was a cohort study (40). Three articles (38–40) found a significant inverse association between screen time and bone health, as evidenced by decreased BMC (measured by DEXA in the proximal femur and the lumbar L1–L4 spine), BMD (measured by DEXA in the hip, femoral neck, and total body), and SOS (measured by QUS at radius and tibia), respectively. One study (37) found no significant association between screen time and bone health (BSI measured in the calcaneus by QUS). That study included the youngest population (preschool and school children aged 2–10 years).

### TV Viewing Time and Bone Health

Seven studies included in this review evaluated the effects of TV viewing time on bone health (39, 41–46). Two of the 7 studies were cohort studies (45, 46) and 5 were cross-sectional studies (39, 41–44). Three reported a significant inverse association between bone health and TV viewing time (39, 41, 44): 2 described a decrease in BMC (measured by DEXA in the proximal femur and the lumbar L1-L4 spine) and 1 a decrease in BSI (measured in the calcaneus by QUS). The participants in the 2 studies that reported a negative association between TV viewing time and BMC were the oldest (18.5–22 years) of all the studies included in this review. Four studies (42, 43, 45, 46) reported no significant effect of TV viewing time on bone health, as determined by total and lumbar BMD and BMC, measured by DEXA (1 study) (42); FI (1 study) (43); and SOS and BUA measured at the mid calcaneus by QUS (2 studies) (45, 46).

### Recreational Computer Time and Bone Health

Six studies included in this review evaluated the effects of recreational computer use on bone health (39, 41, 42, 44–46). Two of the 6 studies were cohort studies (45, 46) and 4 were cross-sectional studies (39, 41, 42, 44). Five articles reported no significant association between bone health and recreational computer use (39, 41, 44–46). Two measured BMC (proximal femur and lumbar L1-L4 spine) by DEXA (39, 44), 2 measured SOS and BUA in the mid-calcaneus by QUS (45, 46), and 1 measured BSI in the calcaneus by QUS (41). Only one study (40) found a negative correlation between computer use and lumbar BMD measured by DEXA in a group of children of normal weight (and between computer use and lumbar total BMC in the overweight group only). However, those authors reported a positive relationship between internet use for non-school purposes and total and lumbar BMD and lumbar BMC.

### Risk-of-Bias Assessment

All the articles included in our review received at least 7 stars in the risk of bias assessment performed using the Newcastle-Ottawa scale, indicating that they were suitable for inclusion in the narrative analysis of the results. Four of these papers (38, 39, 42, 43) received 7 stars, while the remaining 6 (37, 40, 41, 44–46) received 8 stars.

All 10 articles included in the review received 2 stars for comparability between individuals. Following assessment of the risk of bias during the individual selection process, 6 of the 10 articles (37, 40, 41, 44–46) received 4 stars. The other 4 articles (38, 39, 42, 43) received 3 stars: in 3 cases (38, 39, 43) due to the absence of any description of measures taken to mitigate the risk of bias and in 1 case (42) because the study did not exclude individuals with chronic diseases that could interfere with the final result. Regarding screen exposure, all studies received 2 stars, as in all cases screen time was measured based on self-reporting or medical records (Supplementary Table 2).

## Discussion

Recent technological advances have led to an increase in the use of screen-based technologies (screen time) by children and youth. This systematic review of observational studies assesses current evidence on the relationship between screen time and bone status in children and teenagers. The results suggest that total screen time is inversely associated with bone health in both groups. This effect persisted when only weekend screen time was considered. Moreover, we observed a negative correlation between TV viewing time and BMC and BSI, but no significant correlation between recreational computer usage time and bone health.

Environmental and lifestyle factors may markedly influence the achievement of genetic potential peak bone mass. Sedentary time, defined as time spent sitting or lying for extended periods of time, has become a global health concern in recent years (6–11). It is estimated that roughly half of children and youth (47) exceed the maximum screen time of 2 hours per day recommended by public health bodies (20), and even adolescents in the USA exceed 5 hours per day (18). A growing body of evidence associates excessive screen time with numerous deleterious outcomes, including obesity (11, 48), cardiometabolic risk (49), adverse sleep outcomes (13), visual (15) and psychological effects (50) with negative behavioral impacts, and lower self-esteem (51).

The ongoing worldwide coronavirus disease 2019 (COVID-19) pandemic and lockdown have markedly accentuated the trend toward increasing screen time, a consequence of a shift toward online working, educating, and socializing that will likely persist for the foreseeable future (52). This shift has coincided with a concomitant decrease in physical activity resulting from the epidemiological situation and temporary home confinement (53–56). Thus, in the short and medium term we can expect a potential global outbreak of adverse effects linked to excessive screen time. In this context, evaluation of the influence of screen time on bone health is particularly important.

Peak bone mass achieved during youth is the strongest predictor of osteoporosis risk in later life (57). Increased bone mass in childhood and youth is associated with the frequency and intensity of physical activity (58–60) due to the osteogenic effect of exercise. Physical activity mediates different changes beneficial to improve bone mass and promote bone formation (61). Exercise-mechanical loadings are essential stimuli for osteoblast differentiation and mineralization, regulate hormones and cytokines secretion that could play a role in bone metabolism (62), and promote bone angiogenic-osteogenic responses via the modulation of angiogenic mediators and signaling pathways (63, 64). Therefore, the progressive increase in the use of screens in recent years (65), as it increases the inactive time, is expected to have a negative influence on bone status, especially in late childhood and peripubertal years, a critical period for bone accretion.

Total and weekly screen time was negatively correlated with bone mass in 3 (38–40) of the 4 studies that evaluated these parameters. When analyzing TV viewing time this negative correlation was only observed in 50% of the studies included (39, 41, 44), and in 1 study that included males only (45). Only the study published by Vicente-Rodríguez et al. (44) offers data on the relative risk increase of low bone mineral content in relation to the time spent watching television in males, allowing us to calculate the fraction of risk attributable to television time, which corresponds to 85.7%. A notable finding was the absence of a negative association between recreational computer usage time and bone mass (39, 41, 44–46). One possible explanation for this observation is that playing videogames may involve greater energy expenditure, equating to mild-intensity exercise (66, 67), compared with watching TV, which does not increase the resting metabolic rate (68). However, this hypothesis does not explain the positive correlation observed in one study of adolescents between internet use for non-school purposes and BMD (42) unless, as the authors suggest, this use, which probably involves mobile devices, occurs while engaging in active behaviors.

An important aspect to consider is the added physical activity rate in this group of age. It should be noted that although screen time is traditionally associated with sedentary activities, it does not always preclude physical exercise. For example, in the study by Winther et al. (38) 20% of girls and 26% of boys for whom screen time exceeded 4 hours per day also spent more than 4 hours per week playing sports or engaged in high intensity physical activities. Moreover, screen time can also promote physical activity through platforms such as online physical activity classes, exercise applications on mobile devices, and video games with a physical activity component (69, 70). Therefore the individual contributions of sedentarism and physical activity should be distinguished (71). After adjustment by physical activity, some of the studies failed to detect an association between sedentary time and BMC (37, 44). There appears to be a positive association between bone health and a pattern of intermittent periods of sitting punctuated by moderate to vigorous activity (39).

In addition, screen time has been also linked to obesity (11), adiposity (72) and alterations in food and drink consumption, including increased consumption of carbonated drinks (38), sweets, and salty snacks (73–75) that could also influence bone health. Accordingly, Winther et al. found an association between soft drinks consumption and screen time in both sexes (38). However no significant differences between calcium, protein and carbohydrate intake with BUA and SOS were observed (45, 46). Another aspect essential for normal bone development and maintenance is vitamin D whose active form, 1α,25(OH)2D3, is involved in calcium regulation and bone homeostasis. The study in which a multivariate analysis of the relation between sedentary time, nutritional markers, and bone mass was performed, found that the risk of poor bone stiffness was highest in individuals who engaged in low levels of physical activity and had lower serum calcium or 25-OH vitamin D levels (37).

There are well recognized sex differences in bone accrual in terms of the timing of growth and maturation (76). An increased bone turnover was described in males compared to females across adolescence suggesting higher metabolic activity (77). Although screen time was globally higher in males in the reviewed studies (38, 39, 43), a sex-related trend in correlation between bone mass and screen time was observed in only 2 studies: Chastin et al. (39) reported a negative correlation between TV viewing time and femoral BMC in boys and girls and spinal BMC in girls only; and Vicente-Rodriguez et al. (44) reported that TV viewing time was positively associated with the risk of low BMC in males.

The cross-sectional and cohort studies included in this review differ in terms of the method employed to evaluate bone mass, the age range of the children and youth included, and the type of screen time considered (daily or weekly). Four of the studies (38, 39, 42, 44) evaluated BMC by DEXA in the lumbar spine and the neck of the femur, and Winther et al. (38) also evaluated total body BMC by DEXA; 2 studies (37, 41) analyzed BSI by QUS in the calcaneus or the radius and tibia; and the remaining studies analyzed BSI by SOS and BUA in the mid calcaneus (40, 45, 46) and FI (43).

Limitations of this systematic review that should be noted include those inherent to the observational nature of the evaluated studies, as well as the methodological differences in bone measurement and screen time quantification across studies. Likewise, it should be noted the limitations derived from having used only two databases, not having included articles published in languages other than English and Spanish, and not being able to perform meta-analysis due to the heterogeneity of the articles included in the revision. It is possible that negative effects on bone health are progressively accentuated with age, as suggested by the greater negative association between TV viewing time and bone health reported in studies that included older participants. Our findings underscore the need for further studies to assess the long-term effects of screen time on bone status.

## Conclusions

The findings of this systematic review support a negative association between screen time and bone status in children and adolescents, with a different impact of the exposure of the considered technological devices. The studies reviewed revealed a negative correlation between TV viewing time and bone status, but no correlation between recreational computer usage time and bone health.

Osteoporosis is a major public health problem. Bone accretion during childhood and adolescence is a key factor to prevent it. The marked increased in screen time in recent years and its negative association with bone health may lead to an outbreak of this burden worldwide. Strategies promoting lifestyle modifications to achieve peak bone mass and strength should incorporate a multifactorial approach, including promotion of active and conscious use of digital technology.

## Data Availability Statement

The raw data supporting the conclusions of this article will be made available by the authors, without undue reservation.

## Author Contributions

CL, MJ, and MC conceived the study and contributed to the design, methodology, and supervision of the study. PS-P wrote the first draft of the manuscript. MS and MC edited and reviewed the manuscript, and made important intellectual contributions. CL, MJ, and PS-P contributed to data selection and extraction, and the presentation of the results. All authors discussed, revised, and approved the final manuscript.

## Funding

Fundación Instituto de Investigación Sanitaria de Santiago de Compostela.

## Conflict of Interest

The authors declare that the research was conducted in the absence of any commercial or financial relationships that could be construed as a potential conflict of interest.

## Publisher's Note

All claims expressed in this article are solely those of the authors and do not necessarily represent those of their affiliated organizations, or those of the publisher, the editors and the reviewers. Any product that may be evaluated in this article, or claim that may be made by its manufacturer, is not guaranteed or endorsed by the publisher.
