# Supplementary material for: Screen Time and Bone Status in Children and Adolescents: A Systematic Review
Source: Front Pediatr. 2021 Dec 1;9:675214. doi: 10.3389/fped.2021.675214 (PMC8672244; doi:10.3389/fped.2021.675214)
Supplement: Supplementary file 1 [file Data_Sheet_1.PDF]

| **Reason for exclusion** | **n** |
|---|---|
| No screen time data | 45 |
| Study characteristics | 2 |
| No health bone data | 1 |
| Adults | 1 |

**Supplementary Table 1**. Reasons for exclusion of the articles that were selected to read full text
