# Supplementary material for: Screen Time and Bone Status in Children and Adolescents: A Systematic Review
Source: Front Pediatr. 2021 Dec 1;9:675214. doi: 10.3389/fped.2021.675214 (PMC8672244; doi:10.3389/fped.2021.675214)
Supplement: Supplementary file 2 [file Data_Sheet_2.pdf]

	Herrmann et al. (37)	Winther et al. (38)	Chastin et al. (39)	Christoforidis et al. (40)	Cheng et al. (41)	Pelegrini et al. (42)	Rietsch et al. (43)	Vicente et al. (44)	Babaroutsi et al (45)	Babaroutsi et al. (46)
**Selection**										
Representative sample	*	*	*	*	*	*	*	*	*	*
Sample size	*	*	*	*	*	*	*	*	*	*
Non respondents				*	*	*	*		*	*
Ascertainment of the exposure	**	*	*	*	*			**	*	*
**Comparability**										
Control confounding factors	**	**	**	**	**	**	**	**	**	**
**Outcome**										
Assessement of the outcome	*	*	*	*	*	*	*	*	*	*
Statistical test	*	*	*	*	*	*	*	*	*	*
Total	8	7	7	8	8	7	7	8	8	8

Supplementary Table 2. Analysis of the risk of bias by Newcastle Otawa scale
